# Genome sequence data for 61 isolates of Xanthomonas campestris pv. campestris from Brassica crops in Serbia

**DOI:** 10.1099/acmi.0.000870.v3

**Published:** 2024-11-08

**Authors:** Tatjana Popović Milovanović, Shannon Greer, Renata Iličić, Aleksandra Jelušić, Daisy Bown, Mikaeel Hussain, Jamie Harrison, Murray Grant, Joana G. Vicente, David J. Studholme

**Affiliations:** 1 Institute for Plant Protection and Environment, Belgrade, Serbia; 2 School of Life Sciences, University of Warwick, Coventry, UK; 3 Faculty of Agriculture, University of Novi Sad, Novi Sad, Serbia; 4 Institute for Multidisciplinary Research, University of Belgrade, Belgrade, Serbia; 5 Biosciences, University of Exeter, Exeter, UK; 6 Fera Science, York, UK

**Keywords:** black rot, *Brassica napus*, *Brassica oleracea*, oilseed rape, plasmids, race, *Raphanus sativus*

## Abstract

This Technical Resource describes genome sequencing data for 61 isolates of the bacterial pathogen *Xanthomonas campestris* pv. *campestris* collected from *Brassica* and *Raphanus* crops between 2010 and 2021 in Serbia. We present the raw sequencing reads and annotated contig-level genome assemblies and determine the races of ten isolates. The data can be used to test hypotheses and phylogeographic analyses and inform the design of informative molecular markers for population genetics studies. When combined with phenotypic data, they could be used to dissect relationships between genotypes and phenotypes such as host range and virulence. Finally, these genome sequences expand our inventory of plasmids known to reside in this pathogen.

## Data Summary

- BioProject: PRJNA985555 (https://www.ncbi.nlm.nih.gov/bioproject/985555).
- Sequence Read Archive: SRP444796 (https://trace.ncbi.nlm.nih.gov/Traces/?view=study&acc=SRP444796).
- GitHub repository: https://github.com/davidjstudholme/xanthomonas-campestris-serbia.
- DOI for GitHub repository: 10.5281/zenodo.13628631.

The authors confirm all supporting data, code and protocols have been provided within the article or through the public repositories listed above.

## Introduction

*Xanthomonas campestris* pv. *campestris* (*Xcc*) is a bacterial pathogen responsible for causing black rot in cruciferous (*Brassicaceae*) plants, affecting crops such as cabbage, broccoli and cauliflower, oilseed rape and radish [1], and was included among the top ten most important bacterial plant pathogens in molecular plant pathology [2]. Bacteria enter the plant through natural openings or wounds, leading to characteristic V-shaped lesions on leaf margins that progress inward, frequently associated with the blackening of veins. As the disease advances, it disrupts water and nutrient flow, through the production of the mucilaginous sugar xanthan, leading to wilting, stunted growth and even plant death. The bacterium is transmitted via seed and can persist in the field mainly on plant debris, making management and control challenging [1,3, 4]. In Serbia, black rot poses a major threat to the production of cruciferous crops, including oilseed rape [5] and arugula [6].

Genome sequence data can be a useful resource for understanding and managing bacterial plant diseases, including those caused by *Xanthomonas* pathogens [7, 8,11]. Sequencing allows for precise identification of bacterial isolates and their variants, facilitating accurate diagnosis and monitoring of disease outbreaks. It enables the identification of genes responsible for virulence. Knowledge of the pathogen’s genotype can inform the selection and deployment of resistant varieties [12,13]. Genomic data can track the spread and evolution of pathogens over time and across regions [14], informing quarantine and management practices to prevent and control outbreaks [8,15, 16].

Previously, we surveyed *Xcc* populations in Serbia on *Brassica napus* (oilseed rape) and several *Brassica oleracea* crops [5,17, 18]. Those studies used repetitive-element PCR (rep-PCR) and multi-locus sequencing to characterize genetic diversity in the pathogen. Furthermore, they reported results of testing host range and quantification of virulence. Here, we expanded this study, sequencing genomes of a representative subset of those previously characterized isolates augmented with some recently collected isolates. Thus, this dataset offers a valuable resource for future research aimed at understanding the relationship between genotypes and phenotypes in this pathogen.

## Methods

### Bacterial isolates

We previously described collections of *Xcc* isolates from winter oilseed rape (*B. napus*) [10,17] and *B. oleracea* crops [10] in Serbia. One of the isolates, Xc2010, is the same isolate that was described in the first report of *Xcc* causing black rot of oilseed rape in Serbia [5]. In the present study, we included 20 of those *B. napus* isolates and 25 of the previously described *B. oleracea* isolates. We also included a further 14 *B. oleracea* isolates not described in previous studies and 2 *Xcc* isolates from radish. The details of these 61 isolates are provided in Table 1. Bacterial isolates were collected and identified using the methodology described previously [10,17].

**Table 1.** Isolates of *Xcc* whose genomes were sequenced in this study

Isolate name	Crop	Cultivar	Locality	Isolation year	Reference
Xc153 (WHRI 10138)	Broccoli	Unknown	Temerin	2014	[10]
Xc160 (WHRI 10139)	Broccoli	Unknown	Temerin	2014	[10]
Xc163 (WHRI 10141)	Broccoli	Sakata	Futog, South Bačka	2014	[10]
Xc164 (WHRI 10142)	Broccoli	Sakata	Futog, South Bačka	2014	[10]
Xc35 (WHRI 10073)	Cabbage	Futoški	Futog, South Bačka	2014	[10]
Xc39 (WHRI 10074)	Cabbage	Futoški	Futog, South Bačka	2014	[10]
Xc43 (WHRI 10076)	Cabbage	Hybrid	Futog, South Bačka	2014	[10]
Xc51 (WHRI 10079)	Cabbage	Unknown	Temerin	2014	[10]
Xc52 (WHRI 10080)	Cabbage	Unknown	Temerin	2014	[10]
Xc57 (WHRI 10082)	Cabbage	Unknown	Šabac	2014	[10]
Xc58 (WHRI 10083)	Cabbage	Unknown	Šabac	2014	[10]
Xc67 (WHRI 10085)	Cabbage	Unknown	Vrnjačka Banja	2014	[10]
Xc78 (WHRI 10086)	Cabbage	Unknown	Čačak	2014	[10]
Xc1K1/1 (WHRI 10087)	Cabbage	Futoški	Futog, South Bačka	2020	This study
Xc1K2/2 (WHRI 10088)	Cabbage	Futoški	Futog, South Bačka	2020	This study
1Xc10/21T (WHRI 10092)	Cabbage	Unknown	Futog, South Bačka	2021	This study
Xc10/21T (WHRI 10093)	Cabbage	Unknown	Futog, South Bačka	2021	This study
Xc121-1 (WHRI 10100)	Cabbage	Green Boy	Crnjelovo-Bijeljna	2021	This study
Xc121-2 (WHRI 10101)	Cabbage	Green Boy	Crnjelovo-Bijeljna	2021	This study
Xc221-1(WHRI 10090)	Cabbage	Futoški	Futog, South Bačka	2021	This study
Xc221-2 (WHRI 10091)	Cabbage	Futoški	Futog, South Bačka	2021	This study
Xc321-1 (WHRI 10094)	Cabbage	Futoški	Futog, South Bačka	2021	[17]
Xc321-2 (WHRI 10095)	Cabbage	Futoški	Futog, South Bačka	2021	[17]
Xc421-1 (WHRI 10096)	Cabbage	Futoški	Begeč	2021	This study
Xc421-2 (WHRI 10097)	Cabbage	Futoški	Begeč	2021	This study
Xc521-1 (WHRI 10098)	Cabbage	Unknown	Begeč	2021	This study
Xc521-2 (WHRI 10099)	Cabbage	Unknown	Begeč	2021	This study
Xc105 (WHRI 10153)	Cauliflower	Unknown	Futog, South Bačka	2014	[10]
Xc110 (WHRI 10154)	Cauliflower	Unknown	Futog, South Bačka	2014	[10]
Xc111 (WHRI 10156)	Cauliflower	Unknown	Temerin	2014	[10]
Xc115 (WHRI 10157)	Cauliflower	Unknown	Temerin	2014	[10]
Xc142 (WHRI 10159)	Collard greens	Unknown	Temerin	2014	[10]
Xc150 (WHRI 10160)	Collard greens	Unknown	Temerin	2014	[10]
Xc117 (WHRI 10147)	Kale	Unknown	Temerin	2014	[10]
Xc120 (WHRI 10148)	Kale	Unknown	Temerin	2014	[10]
Xc129 (WHRI 10150)	Kale	Clause	Futog, South Bačka	2014	[10]
Xc134 (WHRI 10151)	Kale	Clause	Futog, South Bačka	2014	[10]
Xc173 (WHRI 10144)	Kohlrabi	Unknown	Vrnjačka Banja	2014	[10]
Xc175 (WHRI 10145)	Kohlrabi	Unknown	Vrnjačka Banja	2014	[10]
Xc2010 (WHRI 10102)	Oilseed rape	Slavica	Novi Sad	2010	[5,17]
Xc16 (WHRI 10104)	Oilseed rape	Banaćanka	Kovilj, South Bačka	2014	[10]
Xc20 (WHRI 10106)	Oilseed rape	Unknown	Novi Sad	2014	[10]
Xc25 (WHRI 10107)	Oilseed rape	Unknown	Novi Sad	2014	[10]
Xc7 (WHRI 10103)	Oilseed rape	Banaćanka	Kovilj, South Bačka	2014	[17]
Xc211 (WHRI 10109)	Oilseed rape	Unknown	Ada, Noth Banat	2016	[17]
Xc252 (WHRI 10111)	Oilseed rape	Unknown	Odžaci, West Bačka	2016	[17]
Xc261 (WHRI 10113)	Oilseed rape	Unknown	Nova Crvenka, West Bačka	2016	[17]
Xc272 (WHRI 10115)	Oilseed rape	Triangl	Karavukovo, West Bačka	2016	[17]
Xc292 (WHRI 10117)	Oilseed rape	Unknown	Futog, South Bačka	2016	[17]
Xc302 (WHRI 10119)	Oilseed rape	Unknown	Kovilj, South Bačka	2016	[17]
Xc312 (WHRI 10121)	Oilseed rape	Banaćanka	Rimski Šančevi, South Bačka	2016	[17]
Xc321 (WHRI 10123)	Oilseed rape	Unknown	Gornji Breg, North Banat	2016	[17]
Xc342 (WHRI 10124)	Oilseed rape	Unknown	Bačka Topola, North Bačka	2016	[17]
Xc362 (WHRI 10126)	Oilseed rape	Unknown	Bačka Palanka, South Bačka	2016	[17]
Xc412 (WHRI 10128)	Oilseed rape	SO 14	Rimski Šančevi, South Bačka	2018	[17]
Xc422 (WHRI 10132)	Oilseed rape	Unknown	Rimski Šančevi, South Bačka	2018	[17]
Xc433 (WHRI 10132)	Oilseed rape	Kata	Rimski Šančevi, South Bačka	2018	[17]
Xc442 (WHRI 10134)	Oilseed rape	Nevena	Rimski Šančevi, South Bačka	2018	[17]
Xc452 (WHRI 10136)	Oilseed rape	NS RAS (hybrid)	Rimski Šančevi, South Bačka	2018	[17]
XRE1 (WHRI 10198)	Radish	Unknown	Futog, South Bačka	2019	This study
XRE5 (WHRI 10199)	Radish	Unknown	Futog, South Bačka	2019	This study

### Race typing of bacterial isolates

A subset of *Xcc* isolates from cabbage, oilseed rape and radish were race-typed by inoculating to the *Brassica* differential set described by Vicente *et al.* [1, 19,20]. Briefly, *Xcc* isolates were streaked on King’s B medium [21] and incubated at 28 °C for 48 h prior to inoculation. Bacterial growth was scraped from the plate and resuspended in sterile tap water to give a final suspension with OD_600_ of 0.2 to ensure a consistent inoculum pressure; this OD_600_ corresponds to ~2×10^8^ c.f.u. [19]. Four-week-old plants were inoculated by clipping the secondary veins near the leaf margin with ‘mouse tooth’ forceps that had been wrapped in cotton wool and dipped in the inoculum. The three youngest leaves were inoculated per plant, and two to three plants were inoculated per differential *Brassica* line. Symptoms of infection were scored 2 weeks post-inoculation. Two metrics were scored: the proportion of inoculation points that developed symptoms (PIP) and the size of the largest lesion (LS) on a scale of 0 to 9, where 0=no lesion, 1=small lesion encircling the inoculation point or 3, 5, 7 or 9, which represent, respectively, lesions reaching 25, 50, 75 or 100% of the way to the mid-vein of the leaf. Average PIP and LS were calculated for each differential line from the scores of two to three plants per line and three inoculated leaves per plant. Disease index (DI) was then calculated following the previously described procedure [20]. ‘−’ indicates an incompatible reaction (resistance) where DI<0.1. Weak pathogenesis is indicated by ‘(+)’, where 0.1≥DI<0.7. Susceptibility (compatible reaction) is indicated by ‘+’, where DI≥0.7.

### DNA extraction

Bacteria were grown in King’s B liquid medium [21] at 28 °C and DNA was extracted as described previously [22]. Genomic DNA was extracted from frozen bacterial pellets. Full protocols for bacterial culture and DNA extraction are described at protocols.io via web links https://dx.doi.org/10.17504/protocols.io.5jyl89428v2w/v1 and https://dx.doi.org/10.17504/protocols.io.ewov1nr92gr2/v1.

### Genomic DNA sequencing and assembly

Whole-genome shotgun sequencing was performed on the Illumina NovaSeq 6000 platform to generate pairs of 151-nt sequence reads. Prior to assembly, we performed filtering and trimming of the sequence reads using Trim Galore [23] version 0.6.7, which utilizes Cutadapt [24] version 3.5. The command line was as follows: trim_galore -q 30 --paired reads-file.1.fastq.gz reads-file.2.fastq.gz

We assembled genome sequences using version 0.5.1 of Unicycler [25], which incorporated SPAdes [26] version 4.0.0. The command line used for each assembly was as follows: unicycler-runner.py −1 trimmed-reads-file.1.fastq.gz −2 trimmed-reads-file.2.fastq.gz -o unicycler_output_directory.

Genome assemblies were submitted to GenBank [27] via the National Center for Biotechnology Information (NCBI) Submission Portal [28], after which they were annotated via the NCBI Prokaryotic Genome Annotation Pipeline version 6.5 [29]. Assembly quality was assessed using CheckM version 1.2.2 [30,31].

### Phylogenomics

We generated a phylogenomic tree from genome sequence assemblies using PhaME [32]. PhaME can use several different tree-building software packages; here, we chose FastTree 2 [33], which uses the generalized time-reversible model [34]. To assist rooting the tree, we included the genomes of *X. campestris* pv. *raphani* CFBP 5828R [35] and * X. campestris* pv. *raphani* NCPPB 1946 as an outgroup, which is the pathotype strain of this pathovar [36]. We performed 1000 bootstraps. All command lines and configuration files are available via GitHub at https://github.com/davidjstudholme/xanthomonas-campestris-serbia and Zenodo at https://zenodo.org/doi/10.5281/zenodo.13628631. The resulting phylogenetic tree was rendered as graphics using the web-based Interactive Tree of Life (iTOL) version 6.9.1 [37].

To complement phylogenetic analysis, we also calculated pairwise average nucleotide identities (ANIs) between all the genome assemblies using FastANI [38] version 1.1. The FastANI command line used was as follows: fastANI --ql query_list.txt --rl ref_list.txt -o all-versus-fastANI.short.out -t 6 --visualize –matrix.

### Identification and analysis of putative plasmid sequences

The Unicycler genome assembly pipeline identifies contigs that likely represent circular replicons, such as plasmids [25]. Therefore, we noted all contigs flagged by Unicycler with the string ‘circular=true’ in their description line.

We searched circular contigs for matches against previously reported plasmids via blastn [39] searches using the NCBI web portal [28]. For such searches, we used the following search parameter settings: word size=28, expect value=0.05, hitlist size=100, match score=1, mismatch score=−2, gapcosts=0 and 2.5, low complexity filter=yes, filter string=L;m, genetic code=1 (standard). We searched both the Core Nucleotide database and the Whole-Genome Shotgun Contigs database.

Proksee [40] was used to perform blastn alignments of a plasmid sequence against the genome sequence assemblies and plasmid sequence. For these searches, we used the default settings within Proksee at https://proksee.ca/. No version number was given on that website; the Proksee analysis was performed on 3 and 4 September 2024. blastn searches via Proksee used an expect-value threshold of 0.0001 and genetic code 11 (bacterial). Low-complexity filtering was switched on. No other settings are configurable via the web interface.

We also used EasyFig 2.2.2 to perform and visualize blastn alignments between plasmid sequences [41]. We used the default settings within EasyFig for these alignments. The expect-value threshold was 0.001. Minimum identity and minimum length parameters were both set to zero in EasyFig.

To identify contigs that contain plasmid-like sequences, we performed blastn (version 2.12.0+) searches against each of the 61 genome assemblies, using the CFBP 166 plasmid sequence (GenBank: CP066974.1) as the query. The command line used was as follows:

blastn -db genome.assembly.fasta -query CP066974.1.fna -evalue 0.0000000001 -out blast.output.blastn -outfmt 6

## Results and discussion

### Race typing

In *Xcc*, isolates can be assigned to one of nine races, according to their pathogenicity on a panel of *Brassica* cultivars [1,20]. Table 2 summarizes the results of these pathogenicity tests for ten of the Serbian isolates. On testing against this panel, we found that an isolate from cabbage (Xc321-1) and seven isolates from oilseed rape (Xc2010, Xc7, Xc20, Xc211, Xc252, Xc262 and Xc412) belong to Race 5. The two isolates from radish (XRE1 and XRE5) belong to Race 6. Isolate Xc452 from oilseed rape showed a pattern of pathogenicity that does not match any previously described race, and we have not previously seen isolates displaying this pathogenicity profile. Although isolated from * B. napus* (oilseed rape), Xc452 was non-pathogenic on the *B. napus* doubled haploid COB60 line. However, it showed pathogenicity on Wirosa F_1_ (*B. oleracea*) and PIC1 (*Brassica carinata*). This suggests that the isolate contains an atypical repertoire of virulence and avirulence factors and merits further investigation, including validation of this host range phenotype in other laboratories. Phylogenetically, isolate Xc452 falls within a clade with CFBP 49546, reported to belong to Race 6 [42] isolated from *B. oleracea*. Also in this clade are Serbian isolates Xc442, Xc25 and Xc362, which have not been race-typed. This clade is closely related to a clade that contains isolates reported to belong to Race 3 (CFBP 5683) and Race 5 (CFBP 1713 and CFBP 1712). This close phylogenetic proximity of isolates belonging to different races suggests that the evolution of host range among these pathogens is highly dynamic; detailed genomic comparisons might lead to the identification of genetic determinants of the race phenotype (i.e. host range). Isolate Xc452 is not atypical, sharing between 98.50 and 99.99% ANI with the other 60 genomes (based on FastANI).

**Table 2.** Race typing of 11 *Xcc* isolates from different hosts of origin (cabbage, oilseed rape and radish)

	Isolate (host of origin)										
Brassica line	Xc3211 (cabbage)	Xc2010(oilseed rape)	Xc7(oilseed rape)	Xc20(oilseed rape)	Xc211 (oilseed rape)	Xc252 (oilseed rape)	Xc262 (oilseed rape)	Xc412 (oilseed rape)	Xc452 (oilseed rape)	XRE1(radish)	XRE5(radish)
Wirosa F_1_(*B. oleracea*)	+	+	+	+	+	+	+	+	+	+	+
JRT(*B. rapa*)	+	+	+	+	+	+	+	(+)	(+)	+	+
COB60(*B. napus*)	+	+	(+)	+	+	+	+	+	−	+	+
Seven Top Turnip(*B. rapa*)	(+)	+	+	+ *	+	+	+	(+)*	−	+	+
PIC1(*B. carinata*)	+	+	(+)	+	+	+	+	+	+	+	(+)
FBLM2(*B*. *juncea*)	(+)	(+)	(+)	(+) *	(+) *	(+)	(+)	(+)	−	+	+
Miracle F_1_(*B. oleracea*)	- *	−	−	−	−	−	−	−	−	+	+
SxD1(*B. oleracea*)	−	−	−	−	−	−	−	−	−	+	+
Race assignation	5	5	5	5	5	5	5	5	?	6	6

Symptoms of infection were scored as **+** for compatible interaction (susceptibility), **–** for incompatible interaction (resistance) and (**+**) for weakly pathogenic. Races are defined as previously published [1,20]. An asterisk (*) indicates some variability in results among replicate plants from the same line tested with the same bacterial isolate.

### Genomic DNA sequencing

We performed genome sequencing with the Illumina NovaSeq 6000 platform on the 61 *Xcc* isolates and performed *de novo* assembly using Unicycler. This generated genome assemblies of between 4 855 769 and 5 187 770 bp (see Table 3). The numbers of contigs ranged from 62 to 118, and N_50_ was between 119 005 and 187 482 bp. The G+C content was between 64.93 and 65.26%. We used CheckM to assess the completeness of our genome assemblies by checking for the presence of single-copy marker genes that are expected to be universally present across *Xanthomonas* genomes. Most of the genome assemblies contained 1159 of the 1163 markers, with CheckM reporting a completeness score of 99.89% (see Table 3). All genome assemblies were missing four markers, corresponding to Pfam accessions PF12838.2 (4Fe-4S dicluster domain), PF13429.1 (tetratricopeptide repeat 15), PF05048.8 (periplasmic copper-binding protein repeat) and PF08282.7 (haloacid dehalogenase-like hydrolase repeat). Additionally, the genome assembly for Xc433 was missing PF06903.7 (VirK family). Currently, it is unclear whether these marker genes are genuinely absent from the genomes of these *Xcc* isolates or whether their absence from the genome assemblies represents a failure to correctly assemble these genes from short reads, perhaps due to their containing repetitive sequences. Genome assemblies for isolates Xc110, Xc121-2, 1Xc10/21T and Xc16 were less complete, respectively, missing 10, 10, 20 and 7 markers.

**Table 3.** Summary of genome sequence assembly results. Completeness and contamination were estimated using CheckM [37]. Other metrics were calculated using QUAST [47]. All genome assemblies and raw sequence data are available via BioProject PRJNA985555

GenBank accession	Isolate	Completeness (%)	Contamination (%)	Contigs	Largest contig (bp)	Total length (bp)	GC (%)	N_50_ (bp)
GCA_030362485.2	1Xc10/21T	98.90	0.00	75	314 014	4 990 632	65.06	155 696
GCA_030362505.2	Xc10/21T	99.89	0.00	73	409 526	5 045 758	65.05	177 141
GCA_030361665.2	Xc105	99.89	0.00	74	298 636	5 052 088	65.04	162 313
GCA_030361705.2	Xc110	98.90	0.00	76	329 299	5 020 690	65.02	178 060
GCA_030361725.2	Xc111	99.89	0.00	71	285 017	5 041 637	65.05	149 714
GCA_030361625.2	Xc115	99.89	0.00	76	275 768	5039 121	65.05	149 665
GCA_030361785.2	Xc117	99.89	0.00	78	233 016	5 050 785	65.04	147 257
GCA_030361825.2	Xc120	99.89	0.00	84	422 742	5 047 258	65.04	147 294
GCA_030362325.2	Xc121-1	98.90	0.00	70	348 766	5 011 216	65.03	155 696
GCA_030362385.2	Xc121-2	99.89	0.00	77	392 247	5 041 266	65.05	147 029
GCA_030361745.2	Xc129	99.89	0.00	71	275 780	5 050 891	65.04	155 850
GCA_030361675.2	Xc134	99.89	0.00	71	422 684	5 047 337	65.04	162 305
GCA_030361645.2	Xc142	99.89	0.00	66	431 289	5 045 070	65.05	176 760
GCA_030361585.2	Xc150	99.89	0.00	69	298 690	5 043 137	65.05	162 491
GCA_030361905.2	Xc153	99.89	0.00	76	249 354	5 042 056	65.05	145 690
GCA_030362285.2	Xc16	99.40	0.03	92	296 606	5 025 881	65.08	146 465
GCA_030361945.2	Xc160	99.89	0.00	66	412 023	5 041 939	65.05	184 809
GCA_030361925.2	Xc163	99.89	0.00	77	336 781	5 050 360	65.04	176 816
GCA_030361845.2	Xc164	99.89	0.00	68	392 065	5 041 752	65.05	178 326
GCA_030361765.2	Xc173	99.89	0.00	75	506 247	5 050 923	65.03	155 646
GCA_030361805.2	Xc175	99.89	0.00	74	318 037	5 051 475	65.04	162 603
GCA_030362585.2	Xc1K1/1	99.89	0.00	78	470 146	5 050 918	65.03	165 842
GCA_030362525.2	Xc1K2/2	99.89	0.00	80	328 899	5 040 126	65.05	136 282
GCA_030362235.2	Xc20	99.89	0.00	118	275 871	5 017 256	65.22	162 884
GCA_030362305.2	Xc2010	99.89	0.03	88	601 695	5 034 689	65.11	119 005
GCA_030362225.2	Xc211	99.89	0.07	97	330 923	5 187 770	64.93	161 927
GCA_030362565.2	Xc221-1	99.89	0.00	87	283 455	5 081 384	65.03	146 987
GCA_030362595.2	Xc221-2	99.89	0.00	87	328 866	5 081 559	65.02	146 579
GCA_030362265.2	Xc25	99.89	0.03	79	468 657	4 999 552	65.1	164 319
GCA_030362125.2	Xc252	99.89	0.03	83	602 153	5 121 621	65.03	143 508
GCA_030362185.2	Xc262	99.89	0.03	69	303 136	5 063 515	65.06	178 269
GCA_030362105.2	Xc272	99.89	0.03	98	299 906	5 018 867	65.1	124 593
GCA_030362135.2	Xc292	99.89	0.03	100	340 515	5 103 262	65.04	119 005
GCA_030362155.2	Xc302	99.89	0.00	87	382 450	5 000 095	65.17	162 640
GCA_030362045.2	Xc312	99.89	0.03	81	523 534	5 045 124	65.11	160 784
GCA_030362085.2	Xc321	99.89	0.03	86	523 412	5 019 273	65.1	119 005
GCA_030362465.2	Xc321-1	99.89	0.16	85	301 508	4 989 957	65.13	129 089
GCA_030362435.2	Xc321-2	99.89	0.16	85	301 310	4 990 529	65.13	135 367
GCA_030362025.2	Xc342	99.89	0.03	92	301 766	5 051 896	65.1	145 442
GCA_030362765.2	Xc35	99.89	0.00	98	249 349	5 039 445	65.05	120 676
GCA_030362005.2	Xc362	99.89	0.03	77	302 436	4 982 362	65.12	179 297
GCA_030362745.2	Xc39	99.89	0.00	75	349 028	5 043 533	65.05	155 696
GCA_030362065.2	Xc412	99.89	0.03	62	490 806	5 024 558	65.14	181 474
GCA_030362405.2	Xc421-1	99.89	0.00	73	285 207	5 050 925	65.04	186 596
GCA_030362425.2	Xc421-2	99.89	0.00	71	583 894	5 044 241	65.05	186 737
GCA_030361985.2	Xc422	99.89	0.03	92	523 289	5 093 673	65.06	131 294
GCA_030362725.2	Xc43	99.89	0.00	76	512 463	5 051 703	65.02	168 330
GCA_030361965.2	Xc433	99.64	0.28	78	444 669	5 105 951	65.06	187 329
GCA_030361885.2	Xc442	99.89	0.03	87	349 474	5 050 687	65.04	157 827
GCA_030361865.2	Xc452	99.89	0.03	94	349 942	5 004 522	65.1	153 709
GCA_030362695.2	Xc51	99.89	0.00	74	348 764	5 051 388	65.04	155 696
GCA_030362685.2	Xc52	99.89	0.00	76	349 216	5 049 704	65.04	177 818
GCA_030362315.2	Xc521-1	99.89	0.00	82	348 953	5 041 578	65.05	126 248
GCA_030362335.2	Xc521-2	98.90	0.00	78	374 647	5 011 779	65.03	155 696
GCA_030362645.2	Xc57	99.89	0.00	72	549 940	5 057 520	65.03	177 574
GCA_030362665.2	Xc58	99.89	0.00	73	284 106	5 094 898	65.01	177 574
GCA_030362625.2	Xc67	99.89	0.00	77	275 767	5 045 184	65.04	136 694
GCA_030362205.2	Xc7	99.89	0.03	62	419 228	4 974 376	65.14	187 482
GCA_030362545.2	Xc78	99.89	0.00	76	348 954	5 042 926	65.05	150 991
GCA_030361605.2	XRE1	99.89	0.03	75	298 661	4 857 820	65.26	174 860
GCA_030361545.2	XRE5	99.89	0.03	80	325 203	4 855 769	65.26	161 544

### Phylogenetic positions of Serbian *Xcc* isolates

The 61 genome assemblies shared between 98.64 and 99.30% ANI with the pathotype strain of *Xcc* (ATCC 33913) and only 97.11 and 97.30% with the pathotype strain of *X. campestris* pv. *raphani* (NCPPB 1946). This includes genomes of the Serbian *Xcc* isolates from *Raphinus* as well as those from *Brassica* hosts. These ANI values confirm that the isolates are genetically more similar to *Xcc* than to pv. *raphani* and support their previous identification as *Xcc* based on MLSA and PCR [17,18].

**Fig. 1.**
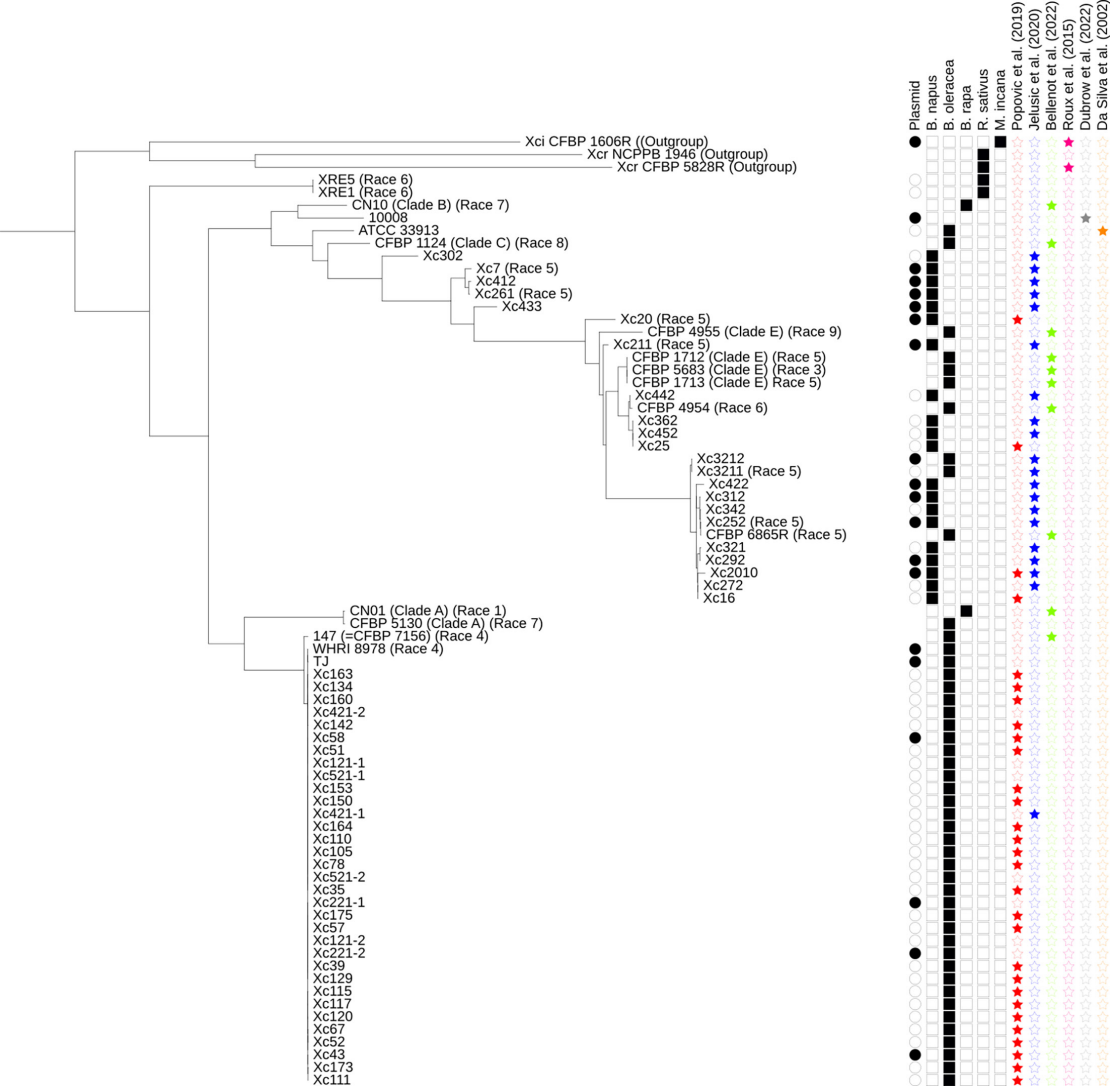
Phylogenetic tree of the 61 *Xcc* isolates from Serbia. Also included are previously published [42] genome sequences representing major clades and races of *Xcc*. The outgroup comprises genomes of pathovars *incanae* and *raphani* [35] and the pathotype strain of pv. *raphani* (NCPPB 1946, unpublished). Also included is a plasmid-containing isolate, 10008 [46]. The coloured stars indicate isolates that were mentioned in previous studies [10,17, 35, 42, 46, 48]. The tree is based on core genome sequences, generated using PhaME [32] and FastTree 2 [33]. The tree was graphically rendered using the iTOL [37].

The phylogenetic relationships of the *Xcc* isolates are summarized in Fig. 1. Thirty-seven isolates fell into a clade that also included the race-4 isolate 147 (= CFBP 7156) from Brazil [42]. This clade is adjacent to two isolates that Guy *et al.* designated as Clade A [43] and includes only isolates from *B. oleracea*, not *B. napus* (oilseed rape).

Eleven isolates share a clade with isolate 6865R, which was isolated from *B. oleracea* in Australia. This clade is adjacent to isolates belonging to Guy’s Clade E [43]. Most of the isolates in this clade were collected from oilseed rape, prior to 2019; however, isolates Xc3211 and Xc3212 were collected from cabbage in 2021, suggesting a possible transmission from oilseed rape to cabbage within Serbia. This clade includes the isolates characterized as highly virulent on the oilseed rape cultivar Zorica [17].

Oilseed rape isolates Xc20, Xc211, Xc442, Xc25, Xc452 and Xc362 are also close to members of Clade E, with Xc442, Xc25, Xc452 and Xc362 being particularly close to CFBP 4594, a Race-6 isolate from cauliflower in Belgium, indicating the host range of the CFBP 6865R clade spans both *B. oleracea* and *B. napus*.

The remaining Serbian oilseed rape isolates are distributed across other earlier-branching clades and the two radish isolates (XRE5 and XRE1) form a distinct clade. Overall, the oilseed rape isolates show a greater diversity than those from *B. oleracea*, which predominantly fall within the clade containing isolate 147, located adjacent to Guy’s Clade A and probably includes only Race 4 isolates. This observation is consistent with previous surveys of genetic diversity using rep-PCR [10,17].

### Several isolates probably contain a plasmid

Plasmids are extra-chromosomal genetic elements that can harbour genes important for pathogenicity and virulence as well as survival in diverse environments [44]. They can play an essential role in adaptation and undergo rapid evolutionary processes [45].

**Fig. 2.**
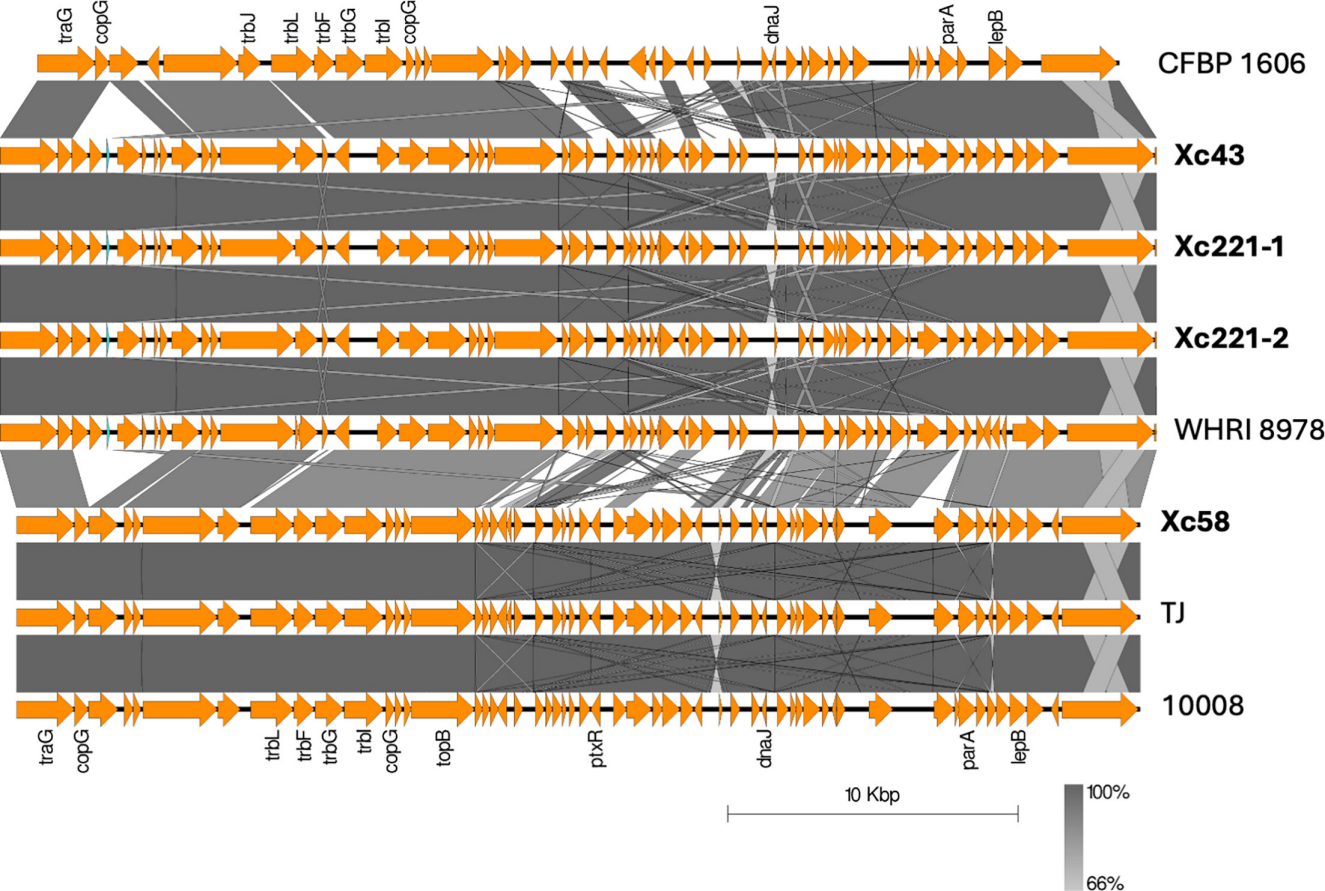
Sequence similarity among plasmids in genomes of Serbian *Xcc* isolates. Easyfig was used to generate and visualize alignments between the nucleotides of putative plasmids [41]. The p38 and p39 sequences are those of contigs that Unicycler identified as circular. Their accession numbers are JAUBNN020000033.1, JAUBNE020000039.1, JAUBND020000035.1 and JAUBNJ020000033.1. Also included are plasmid sequences from *X. campestris* pv. *incanae* CFBP 1606 with accession number CP066924.1 [35], from *Xcc* WHRI 8978 with accession number CP142010.1 (unpublished) and *Xcc* isolates TJ and 10008 with accession numbers CP142010.1 (unpublished) and CP066974.1 [46].

The Unicycler genome assembly pipeline is able to identify contigs that represent circular DNA molecules, such as plasmids [25]. Four of the 61 genome assemblies each contained a contig that Unicycler identified as being circular. The circular sequence from isolate Xc58 was 38 763 bp long and is identical to previously sequenced plasmids from *Xcc* isolates TJ (unpublished) and 10 008 [46]. Isolates Xc43, Xc221-1 and Xc221-2 each shared an identical sequence of 39 903 bp, encoding 99.68% nucleotide identity over 84% of its length with an unnamed plasmid (CP066924.1) from *X. campestris* pv. *incanae* CFPB R [35]. This suggests the existence of two plasmids, which we annotate as p38 and p39. These two plasmids are somewhat distantly related to each other, sharing 88.05% sequence identity over 78% of plasmid p39. Sequence alignments between these plasmids are shown in Fig. 2.

**Fig. 3.**
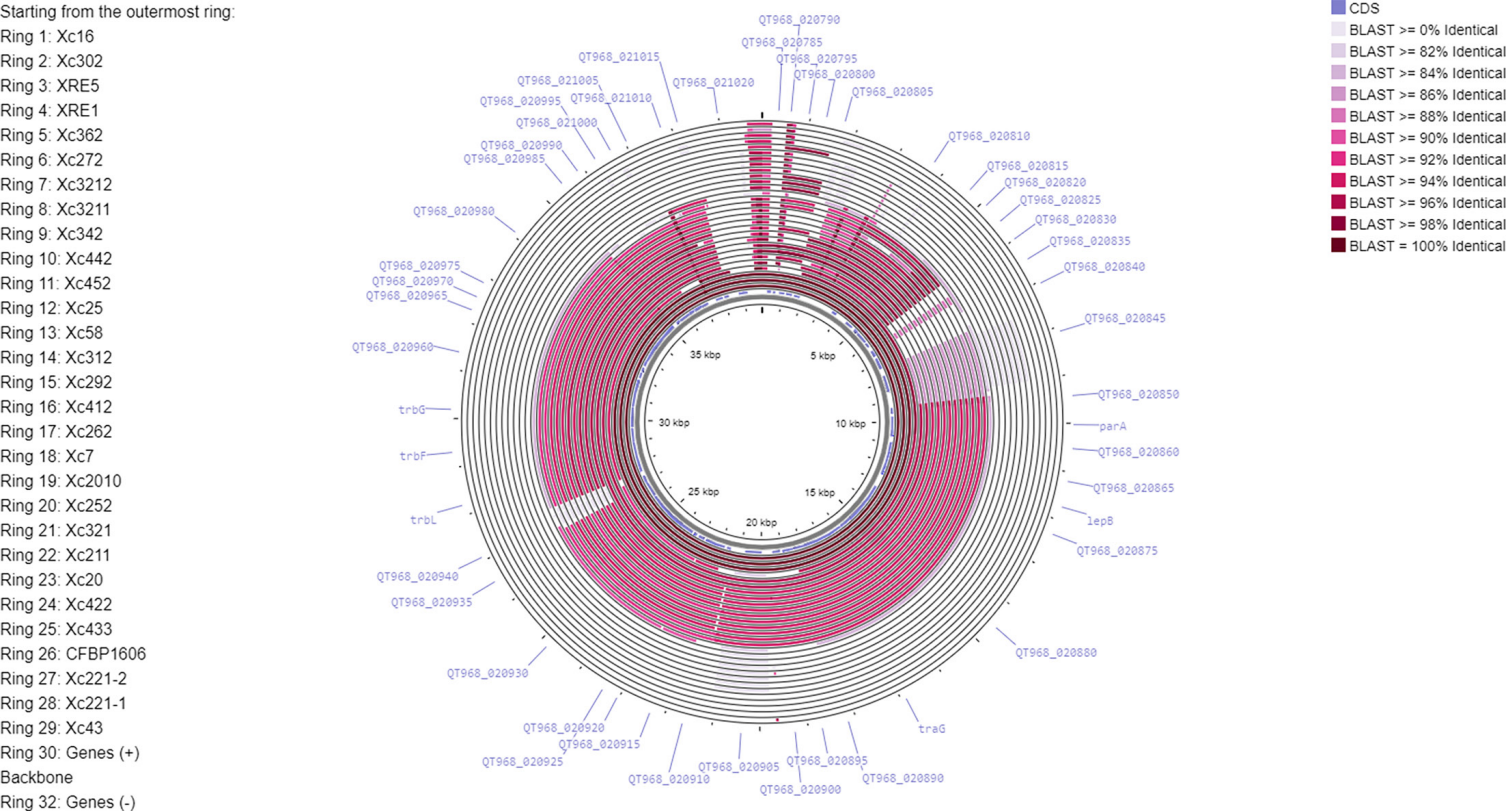
Plasmid-like sequences in the genomes of Serbian *Xcc* isolates. The sequence of circular contig JAUBMB020000033.1 from isolate Xc43 is represented as a circle. Sequence similarity to genome sequences, identified through blastn searches, is indicated by the colouring of concentric circular tracks. The blastn searches were performed and visualized using Proksee web server [40] using default settings.

Several other Serbian isolates shared sequence similarity with plasmids p38 and p39, although sequence conservation did not extend across their entire lengths and Unicycler did not identify the contigs as circular. The lack of circularity may simply reflect the limitations of *de novo* genome assembly based on only short sequence reads and does not preclude the possibility that those isolates do indeed contain plasmids. Plasmid sequences may be fragmented across two or more separate contigs; that would be consistent with the pattern of blastn hits seen in Table S1. The results of aligning genome sequences against plasmid p39 are summarized in Fig. 3 and Table S1. For most isolates, the sequence similarity does not extend over the full length of p39, indicating that these putative plasmids are not identical to p39. The pattern of sequence conservation is also different in most isolates compared with Xc58, further indicating that they do not contain a plasmid identical to p38. Nevertheless, these results (Fig. 3) suggest that isolates Xc422, Xc433, Xc292, Xc312, Xc2010, Xc20, Xc211, Xc252, Xc321, Xc412 and Xc262 contain plasmids sharing extensive sequence similarity to p38 and p39. Other isolates show sequence similarity that is confined to just a few genes, and there is no clear indication of a plasmid location for these. The genome assemblies presented here (and the accompanying raw sequence reads) may be useful for inclusion in future studies of dynamics of plasmid evolution and dissemination in *X. campestris*.

An initial inspection of the annotated sequences of plasmids p38 and p39 reveals genes for conjugal transfer and genes encoding hypothetical proteins of unknown function. Nothing in the annotation indicates virulence factors or antimicrobial resistance.

### Conclusion

This Technical Resource describes the genome sequencing data for 59 isolates of the plant pathogen *Xcc* collected from *Brassica* crops and a further 2 *Xcc* isolates from *Raphanus*, obtained between 2010 and 2021 in Serbia. This study complements and enhances previous studies based on MLSA and rep-PCR [10,17]. Serbian isolates from *B. napus* mainly belong to Race 5 and were included in a clade of diverse isolates that did not include Race 4 isolates. On the other hand, isolates from * B. oleracea* included members of several races and clades but mainly fell within a clade that might be uniformly Race 4. Future phylogeographic analysis of this dataset integrated with genomic data from other geographic regions may provide insights into the spread of specific genotypes across national borders, for example, via international trade in seeds. Several Serbian isolates from different clades and race types contained/accommodated a plasmid, and therefore, these genome sequences expanded our inventory of plasmids known to reside in *Xcc*. The data can be used to test hypotheses about the emergence and population history of this pathogen in Serbia [10,17] and to inform the design of informative molecular markers for future population genetics studies.

## supplementary material

10.1099/acmi.0.000870.v3 Fig. S1.

## Abbreviations

ANI: average nucleotide identities
DI: disease index
iTOL: interactive tree of life
LS: largest lesion
MLSA: multi-locus sequence analysis
NCBI: National Center for Biotechnology Information
PIP: proportion of inoculation points
rep-PCR: repetitive element PCR
*Xcc*: *Xanthomonas campestris* pv. *campestris*
