# Supplementary material for: Genome sequence data for 61 isolates of Xanthomonas campestris pv. campestris from Brassica crops in Serbia
Source: Access Microbiol. 2024 Nov 8;6(11):000870.v3. doi: 10.1099/acmi.0.000870.v3 (PMC11653112; doi:10.1099/acmi.0.000870.v3)
Supplement: Fig. S1. [file acmi-6-00870-s001.pdf]

Supplementary Table 1. Contigs in the Serbian *Xcc* genome sequences that share sequence similarity with the plasmid from isolate CFBP 1606 (GenBank: CP066974.1).

Isolate	GenBank accession	Sequence identity (%)	Length of BLASTN alignment (bp)	Start	End
Xc16	JAUBMQ020000069.1	88.85	574	654	1,226
Xc20	JAUBMP020000038.1	98.32	119	1,943	1,825
Xc20	JAUBMP020000038.1	95.88	97	1,938	1,843
Xc20	JAUBMP020000038.1	95.59	68	1,573	1,506
Xc20	JAUBMP020000071.1	95.35	129	4,264	4,136
Xc20	JAUBMP020000071.1	94.96	119	3,623	3,505
Xc20	JAUBMP020000071.1	94.92	118	3,622	3,505
Xc20	JAUBMP020000071.1	94.85	97	4,259	4,164
Xc20	JAUBMP020000038.1	93.42	1,033	1,943	911
Xc20	JAUBMP020000038.1	91.16	3,926	16,504	12,581
Xc20	JAUBMP020000071.1	90.09	232	3,479	3,248
Xc20	JAUBMP020000038.1	90.07	987	17,562	16,576
Xc20	JAUBMP020000086.1	88.63	589	1,675	1,088
Xc20	JAUBMP020000038.1	87.86	7,635	29,415	21,814
Xc20	JAUBMP020000038.1	87.70	374	34,497	34,130
Xc20	JAUBMP020000095.1	87.52	537	652	1,181
Xc20	JAUBMP020000038.1	86.11	1,785	34,002	32,235
Xc20	JAUBMP020000071.1	85.36	1,284	4,787	3,522
Xc20	JAUBMP020000071.1	85.28	2,343	2,929	625
Xc20	JAUBMP020000038.1	84.80	1,474	31,467	29,999
Xc20	JAUBMP020000038.1	84.35	7,889	11,742	3,913
Xc20	JAUBMP020000038.1	84.08	1,332	20,432	19,117
Xc20	JAUBMP020000067.1	78.75	687	4,998	5,679
Xc20	JAUBMP020000038.1	74.88	804	26,278	25,489
Xc2010	JAUBMS020000053.1	99.16	119	4,738	4,620
Xc2010	JAUBMS020000041.1	98.32	119	2,462	2,344
Xc2010	JAUBMS020000053.1	98.28	232	3,450	3,219
Xc2010	JAUBMS020000053.1	96.74	92	4,733	4,643
Xc2010	JAUBMS020000041.1	95.88	97	2,457	2,362
Xc2010	JAUBMS020000053.1	95.00	120	4,096	3,977
Xc2010	JAUBMS020000053.1	94.49	127	4,095	3,969
Xc2010	JAUBMS020000041.1	94.05	1,025	2,462	1,438
Xc2010	JAUBMS020000041.1	90.73	3,927	17,026	13,101
Xc2010	JAUBMS020000041.1	90.34	973	18,068	17,096
Xc2010	JAUBMS020000067.1	88.64	581	1,666	1,087

Xc2010	JAUBMS020000041.1	88.01	7,634	32,245	24,643
Xc2010	JAUBMS020000041.1	87.70	374	37,326	36,959
Xc2010	JAUBMS020000053.1	87.12	1,491	5,056	3,593
Xc2010	JAUBMS020000053.1	87.09	581	7,077	6,499
Xc2010	JAUBMS020000041.1	86.03	1,782	36,831	35,065
Xc2010	JAUBMS020000053.1	85.40	2,343	2,900	592
Xc2010	JAUBMS020000041.1	84.80	1,474	34,297	32,829
Xc2010	JAUBMS020000041.1	84.40	1,327	23,272	21,959
Xc2010	JAUBMS020000041.1	84.27	7,889	12,262	4,432
Xc2010	JAUBMS020000041.1	74.93	750	29,058	28,318
Xc211	JAUBMN020000040.1	98.32	119	32,400	32,518
Xc211	JAUBMN020000040.1	95.88	97	32,405	32,500
Xc211	JAUBMN020000066.1	95.35	129	2,429	2,557
Xc211	JAUBMN020000066.1	94.96	119	3,070	3,188
Xc211	JAUBMN020000066.1	94.85	97	2,434	2,529
Xc211	JAUBMN020000073.1	94.15	188	1,470	1,283
Xc211	JAUBMN020000040.1	94.15	1,025	32,400	33,424
Xc211	JAUBMN020000066.1	92.91	127	3,062	3,188
Xc211	JAUBMN020000073.1	92.70	274	3,221	2,948
Xc211	JAUBMN020000040.1	91.16	3,926	17,839	21,762
Xc211	JAUBMN020000066.1	90.09	232	3,214	3,445
Xc211	JAUBMN020000040.1	90.07	987	16,781	17,767
Xc211	JAUBMN020000066.1	89.06	329	1	323
Xc211	JAUBMN020000040.1	87.86	7,635	4,928	12,529
Xc211	JAUBMN020000040.1	87.62	218	2	213
Xc211	JAUBMN020000040.1	86.26	1,769	341	2,093
Xc211	JAUBMN020000090.1	85.58	208	652	858
Xc211	JAUBMN020000066.1	85.28	2,343	3,764	6,068
Xc211	JAUBMN020000066.1	85.14	1,299	1,891	3,171
Xc211	JAUBMN020000040.1	84.80	1,474	2,876	4,344
Xc211	JAUBMN020000039.1	84.52	1,098	35,941	37,028
Xc211	JAUBMN020000040.1	84.35	7,889	22,601	30,430
Xc211	JAUBMN020000040.1	84.08	1,332	13,911	15,226
Xc211	JAUBMN020000039.1	82.53	372	3,088	3,450
Xc211	JAUBMN020000040.1	79.40	199	32,639	32,829
Xc211	JAUBMN020000039.1	76.10	1,042	1,963	2,963
Xc211	JAUBMN020000040.1	74.91	801	8,050	8,836
Xc221-1	JAUBNE020000039.1	98.32	119	36,887	36,769
Xc221-1	JAUBNE020000039.1	97.48	119	31,254	31,136
Xc221-1	JAUBNE020000039.1	97.46	118	31,253	31,136
Xc221-1	JAUBNE020000039.1	95.88	97	36,882	36,787

Xc221-1	JAUBNE020000039.1	95.35	129	31,895	31,767
Xc221-1	JAUBNE020000039.1	94.85	97	31,890	31,795
Xc221-1	JAUBNE020000039.1	94.12	1,122	36,887	35,766
Xc221-1	JAUBNE020000039.1	90.81	979	12,383	11,407
Xc221-1	JAUBNE020000039.1	90.70	4,044	11,334	7,293
Xc221-1	JAUBNE020000039.1	90.52	232	31,110	30,879
Xc221-1	JAUBNE020000039.1	88.83	1,137	29,230	28,099
Xc221-1	JAUBNE020000039.1	88.05	7,641	23,400	15,791
Xc221-1	JAUBNE020000039.1	87.18	554	34,535	33,989
Xc221-1	JAUBNE020000039.1	86.61	6,771	6,737	1
Xc221-1	JAUBNE020000039.1	85.51	1,284	32,418	31,153
Xc221-1	JAUBNE020000039.1	85.33	593	27,983	27,407
Xc221-1	JAUBNE020000039.1	84.12	1,329	14,417	13,105
Xc221-1	JAUBNE020000039.1	84.09	886	24,797	23,920
Xc221-1	JAUBNE020000039.1	83.73	1,948	27,006	25,076
Xc221-1	JAUBNE020000039.1	83.03	1,367	30,566	29,231
Xc221-1	JAUBNE020000039.1	82.34	1,053	39,903	38,866
Xc221-1	JAUBNE020000039.1	78.89	199	36,648	36,458
Xc221-1	JAUBNE020000039.1	74.75	804	20,255	19,466
Xc221-2	JAUBND020000035.1	98.32	119	17,680	17,798
Xc221-2	JAUBND020000035.1	97.48	119	23,313	23,431
Xc221-2	JAUBND020000035.1	95.88	97	17,685	17,780
Xc221-2	JAUBND020000035.1	95.35	129	22,672	22,800
Xc221-2	JAUBND020000035.1	95.28	127	23,305	23,431
Xc221-2	JAUBND020000035.1	94.85	97	22,677	22,772
Xc221-2	JAUBND020000035.1	94.12	1,122	17,680	18,801
Xc221-2	JAUBND020000035.1	90.81	979	2,281	3,257
Xc221-2	JAUBND020000035.1	90.70	4,044	3,330	7,371
Xc221-2	JAUBND020000035.1	90.52	232	23,457	23,688
Xc221-2	JAUBND020000035.1	88.83	1,137	25,334	26,465
Xc221-2	JAUBND020000035.1	88.05	7,641	31,167	38,776
Xc221-2	JAUBND020000035.1	87.52	545	20,029	20,566
Xc221-2	JAUBND020000035.1	86.02	7,826	7,924	15,701
Xc221-2	JAUBND020000035.1	85.33	593	26,584	27,160
Xc221-2	JAUBND020000035.1	85.30	1,299	22,134	23,414
Xc221-2	JAUBND020000035.1	84.12	1,329	247	1,559
Xc221-2	JAUBND020000035.1	84.09	886	29,770	30,647
Xc221-2	JAUBND020000035.1	83.85	1,932	27,561	29,476
Xc221-2	JAUBND020000035.1	83.03	1,367	24,001	25,336
Xc221-2	JAUBND020000035.1	78.89	199	17,919	18,109
Xc221-2	JAUBND020000035.1	74.78	801	34,297	35,083

Xc25	JAUBMO020000035.1	95.53	179	385	563
Xc25	JAUBMO020000067.1	89.00	582	421	1,001
Xc25	JAUBMO020000064.1	88.85	574	1,099	527
Xc25	JAUBMO020000035.1	84.52	1,098	5,713	4,626
Xc25	JAUBMO020000035.1	82.92	363	38,557	38,204
Xc25	JAUBMO020000035.1	82.58	534	3,990	3,463
Xc25	JAUBMO020000035.1	76.02	1,055	39,691	38,679
Xc252	JAUBMM020000048.1	98.32	119	16,321	16,203
Xc252	JAUBMM020000048.1	96.90	129	4,097	3,969
Xc252	JAUBMM020000048.1	96.74	92	4,092	4,002
Xc252	JAUBMM020000048.1	95.88	97	16,316	16,221
Xc252	JAUBMM020000048.1	94.96	119	3,456	3,338
Xc252	JAUBMM020000048.1	94.92	118	3,455	3,338
Xc252	JAUBMM020000048.1	92.02	451	16,321	15,871
Xc252	JAUBMM020000069.1	91.82	281	1,367	1,087
Xc252	JAUBMM020000038.1	91.14	3,926	17,839	21,762
Xc252	JAUBMM020000048.1	90.56	339	15,870	15,532
Xc252	JAUBMM020000048.1	90.52	232	3,312	3,081
Xc252	JAUBMM020000038.1	90.07	987	16,781	17,767
Xc252	JAUBMM020000048.1	88.28	128	15,344	15,217
Xc252	JAUBMM020000038.1	87.90	7,634	4,927	12,529
Xc252	JAUBMM020000038.1	87.62	218	2	213
Xc252	JAUBMM020000048.1	86.98	576	5,669	5,095
Xc252	JAUBMM020000038.1	86.20	1,768	341	2,092
Xc252	JAUBMM020000048.1	85.28	2,343	2,774	464
Xc252	JAUBMM020000048.1	85.28	1,284	4,620	3,355
Xc252	JAUBMM020000038.1	84.80	7,883	22,601	30,427
Xc252	JAUBMM020000038.1	84.80	1,474	2,875	4,343
Xc252	JAUBMM020000038.1	84.10	1,333	13,911	15,226
Xc252	JAUBMM020000075.1	82.47	154	153	1
Xc252	JAUBMM020000048.1	79.40	199	16,082	15,892
Xc252	JAUBMM020000038.1	74.41	801	8,050	8,836
Xc262	JAUBML020000043.1	99.16	119	10,356	10,474
Xc262	JAUBML020000043.1	98.39	62	12,019	12,080
Xc262	JAUBML020000034.1	98.32	119	4,526	4,408
Xc262	JAUBML020000043.1	97.73	44	12,019	12,062
Xc262	JAUBML020000043.1	97.10	69	12,019	12,087
Xc262	JAUBML020000043.1	96.74	92	10,361	10,451
Xc262	JAUBML020000043.1	96.43	644	12,059	12,702
Xc262	JAUBML020000034.1	95.88	97	4,521	4,426
Xc262	JAUBML020000043.1	94.62	130	3,999	4,128

Xc262	JAUBML020000034.1	94.15	1,025	4,526	3,502
Xc262	JAUBML020000034.1	91.16	3,926	19,084	15,161
Xc262	JAUBML020000034.1	90.07	987	20,142	19,156
Xc262	JAUBML020000043.1	88.08	1,032	10,038	11,060
Xc262	JAUBML020000043.1	87.97	582	7,987	8,566
Xc262	JAUBML020000034.1	87.84	7,630	31,995	24,394
Xc262	JAUBML020000034.1	87.70	374	37,076	36,709
Xc262	JAUBML020000034.1	86.04	1,784	36,581	34,815
Xc262	JAUBML020000043.1	85.58	2,344	13,015	15,320
Xc262	JAUBML020000034.1	84.80	1,474	34,047	32,579
Xc262	JAUBML020000034.1	84.75	7,883	14,322	6,496
Xc262	JAUBML020000034.1	84.01	1,332	23,012	21,697
Xc262	JAUBML020000034.1	79.40	199	4,287	4,097
Xc262	JAUBML020000034.1	74.75	804	28,858	28,069
Xc272	JAUBMK020000055.1	94.15	188	10,288	10,101
Xc272	JAUBMK020000085.1	89.27	559	1,085	527
Xc272	JAUBMK020000084.1	89.25	558	2	559
Xc272	JAUBMK020000055.1	84.52	1,098	4,960	6,047
Xc272	JAUBMK020000042.1	82.92	363	27,888	27,535
Xc272	JAUBMK020000055.1	82.67	531	6,683	7,207
Xc272	JAUBMK020000042.1	76.02	1,055	29,022	28,010
Xc292	JAUBMJ020000066.1	99.16	119	5,210	5,092
Xc292	JAUBMJ020000041.1	98.32	119	4,523	4,405
Xc292	JAUBMJ020000066.1	98.28	232	3,922	3,691
Xc292	JAUBMJ020000066.1	96.74	92	5,205	5,115
Xc292	JAUBMJ020000041.1	95.88	97	4,518	4,423
Xc292	JAUBMJ020000066.1	95.00	120	4,568	4,449
Xc292	JAUBMJ020000066.1	94.49	127	4,567	4,441
Xc292	JAUBMJ020000041.1	94.15	1,025	4,523	3,499
Xc292	JAUBMJ020000098.1	91.27	275	604	331
Xc292	JAUBMJ020000041.1	90.73	3,927	19,099	15,174
Xc292	JAUBMJ020000041.1	90.34	973	20,141	19,169
Xc292	JAUBMJ020000081.1	88.68	574	652	1,224
Xc292	JAUBMJ020000041.1	88.01	7,634	34,318	26,716
Xc292	JAUBMJ020000041.1	87.62	218	39,243	39,032
Xc292	JAUBMJ020000066.1	87.06	1,491	5,528	4,065
Xc292	JAUBMJ020000041.1	86.04	1,784	38,904	37,138
Xc292	JAUBMJ020000041.1	85.95	7,874	14,335	6,493
Xc292	JAUBMJ020000066.1	85.23	2,342	3,372	1,072
Xc292	JAUBMJ020000041.1	84.80	1,474	36,370	34,902
Xc292	JAUBMJ020000041.1	84.40	1,327	25,345	24,032

Xc292	JAUBMJ020000041.1	79.40	199	4,284	4,094
Xc292	JAUBMJ020000041.1	74.93	750	31,131	30,391
Xc302	JAUBMI020000064.1	85.65	1,324	968	2,284
Xc312	JAUBMH020000036.1	99.16	119	41,197	41,315
Xc312	JAUBMH020000036.1	98.28	232	42,485	42,716
Xc312	JAUBMH020000036.1	96.74	92	41,202	41,292
Xc312	JAUBMH020000036.1	95.00	120	41,839	41,958
Xc312	JAUBMH020000036.1	94.00	100	34,301	34,396
Xc312	JAUBMH020000036.1	94.00	100	34,301	34,396
Xc312	JAUBMH020000036.1	92.65	136	41,831	41,966
Xc312	JAUBMH020000036.1	91.34	127	34,305	34,426
Xc312	JAUBMH020000036.1	90.83	709	34,623	35,325
Xc312	JAUBMH020000036.1	90.69	3,933	20,482	24,413
Xc312	JAUBMH020000036.1	90.19	979	19,440	20,418
Xc312	JAUBMH020000060.1	88.85	574	653	1,225
Xc312	JAUBMH020000036.1	88.01	7,634	5,269	12,871
Xc312	JAUBMH020000036.1	87.73	587	38,852	39,436
Xc312	JAUBMH020000036.1	87.70	374	188	555
Xc312	JAUBMH020000036.1	87.06	1,491	40,879	42,342
Xc312	JAUBMH020000036.1	86.20	1,768	683	2,434
Xc312	JAUBMH020000036.1	85.23	2,342	43,035	45,335
Xc312	JAUBMH020000036.1	84.80	1,474	3,217	4,685
Xc312	JAUBMH020000036.1	84.40	1,327	14,242	15,555
Xc312	JAUBMH020000036.1	83.37	8,599	25,252	33,770
Xc312	JAUBMH020000036.1	75.10	731	8,456	9,178
Xc321	JAUBMG020000039.1	98.32	119	32,739	32,857
Xc321	JAUBMG020000039.1	98.28	232	45,396	45,627
Xc321	JAUBMG020000039.1	96.70	91	44,125	44,214
Xc321	JAUBMG020000039.1	96.12	129	44,119	44,247
Xc321	JAUBMG020000039.1	95.88	97	32,744	32,839
Xc321	JAUBMG020000039.1	95.08	61	37,615	37,675
Xc321	JAUBMG020000039.1	95.00	120	44,750	44,869
Xc321	JAUBMG020000039.1	94.15	1,025	32,739	33,763
Xc321	JAUBMG020000039.1	93.37	181	37,427	37,607
Xc321	JAUBMG020000039.1	92.65	136	44,742	44,877
Xc321	JAUBMG020000039.1	91.14	3,926	18,181	22,104
Xc321	JAUBMG020000039.1	90.07	987	17,123	18,109
Xc321	JAUBMG020000064.1	88.85	574	652	1,224
Xc321	JAUBMG020000039.1	87.90	7,634	5,269	12,871
Xc321	JAUBMG020000039.1	87.70	374	188	555
Xc321	JAUBMG020000039.1	86.20	1,768	683	2,434

Xc321	JAUBMG020000039.1	85.26	2,347	45,946	48,258
Xc321	JAUBMG020000039.1	84.80	1,474	3,217	4,685
Xc321	JAUBMG020000039.1	84.79	7,883	22,943	30,769
Xc321	JAUBMG020000039.1	84.46	1,718	43,581	45,253
Xc321	JAUBMG020000039.1	84.08	1,332	14,253	15,568
Xc321	JAUBMG020000039.1	81.98	1,587	38,110	39,671
Xc321	JAUBMG020000039.1	79.40	199	32,978	33,168
Xc321	JAUBMG020000039.1	74.41	801	8,392	9,178
Xc321-1	JAUBNA020000040.1	95.53	179	952	1,130
Xc321-1	JAUBNA020000071.1	89.27	559	1	559
Xc321-1	JAUBNA020000070.1	89.03	565	1,084	520
Xc321-1	JAUBNA020000040.1	84.52	1,098	6,280	5,193
Xc321-1	JAUBNA020000040.1	82.92	363	39,124	38,771
Xc321-1	JAUBNA020000040.1	82.58	534	4,557	4,030
Xc321-1	JAUBNA020000040.1	76.02	1,055	40,258	39,246
Xc321-2	JAUBMZ020000040.1	95.53	179	385	563
Xc321-2	JAUBMZ020000073.1	89.27	559	1	559
Xc321-2	JAUBMZ020000072.1	89.03	565	1,084	520
Xc321-2	JAUBMZ020000040.1	84.52	1,098	5,713	4,626
Xc321-2	JAUBMZ020000040.1	82.92	363	38,557	38,204
Xc321-2	JAUBMZ020000040.1	82.58	534	3,990	3,463
Xc321-2	JAUBMZ020000040.1	76.02	1,055	39,691	38,679
Xc342	JAUBMF020000041.1	95.53	179	385	563
Xc342	JAUBMF020000080.1	88.85	574	23	595
Xc342	JAUBMF020000079.1	88.64	581	1,099	520
Xc342	JAUBMF020000041.1	84.52	1,098	5,713	4,626
Xc342	JAUBMF020000055.1	82.92	363	6,943	6,590
Xc342	JAUBMF020000041.1	82.58	534	3,990	3,463
Xc342	JAUBMF020000055.1	76.02	1,055	8,077	7,065
Xc362	JAUBME020000058.1	88.80	589	1,674	1,087
Xc412	JAUBMD020000032.1	98.32	119	32,875	32,993
Xc412	JAUBMD020000036.1	98.32	119	7,050	6,932
Xc412	JAUBMD020000036.1	96.67	120	6,411	6,292
Xc412	JAUBMD020000036.1	96.06	127	6,410	6,284
Xc412	JAUBMD020000032.1	95.88	97	32,880	32,975
Xc412	JAUBMD020000036.1	95.88	97	7,045	6,950
Xc412	JAUBMD020000036.1	94.62	130	12,409	12,280
Xc412	JAUBMD020000032.1	94.15	1,025	32,875	33,899
Xc412	JAUBMD020000032.1	91.16	3,926	18,317	22,240
Xc412	JAUBMD020000032.1	90.07	987	17,259	18,245
Xc412	JAUBMD020000036.1	89.48	1,740	3,593	1,858

Xc412	JAUBMD020000032.1	87.88	7,633	5,406	13,007
Xc412	JAUBMD020000036.1	87.71	586	8,421	7,839
Xc412	JAUBMD020000032.1	87.70	374	324	691
Xc412	JAUBMD020000036.1	86.84	1,201	7,369	6,200
Xc412	JAUBMD020000032.1	86.15	1,769	819	2,571
Xc412	JAUBMD020000032.1	84.80	1,474	3,354	4,822
Xc412	JAUBMD020000032.1	84.75	7,883	23,079	30,905
Xc412	JAUBMD020000036.1	84.73	982	4,723	3,755
Xc412	JAUBMD020000032.1	84.08	1,332	14,389	15,704
Xc412	JAUBMD020000032.1	79.40	199	33,114	33,304
Xc412	JAUBMD020000036.1	77.90	285	3,669	3,389
Xc412	JAUBMD020000032.1	74.78	801	8,528	9,314
Xc422	JAUBMC020000052.1	99.16	119	4,737	4,619
Xc422	JAUBMC020000040.1	98.32	119	31,670	31,788
Xc422	JAUBMC020000052.1	98.28	232	3,449	3,218
Xc422	JAUBMC020000052.1	96.74	92	4,732	4,642
Xc422	JAUBMC020000040.1	95.88	97	31,675	31,770
Xc422	JAUBMC020000052.1	95.00	120	4,095	3,976
Xc422	JAUBMC020000052.1	94.49	127	4,094	3,968
Xc422	JAUBMC020000040.1	94.15	1,025	31,670	32,694
Xc422	JAUBMC020000040.1	90.90	4,045	17,328	21,370
Xc422	JAUBMC020000040.1	90.50	979	16,279	17,255
Xc422	JAUBMC020000065.1	88.64	581	1,666	1,087
Xc422	JAUBMC020000040.1	87.74	7,634	5,269	12,871
Xc422	JAUBMC020000040.1	87.70	374	188	555
Xc422	JAUBMC020000052.1	87.12	1,491	5,055	3,592
Xc422	JAUBMC020000052.1	87.09	581	7,076	6,498
Xc422	JAUBMC020000040.1	86.15	7,824	21,923	29,700
Xc422	JAUBMC020000040.1	86.07	1,766	683	2,434
Xc422	JAUBMC020000052.1	85.40	2,343	2,899	591
Xc422	JAUBMC020000040.1	84.80	1,474	3,217	4,685
Xc422	JAUBMC020000040.1	84.12	1,329	14,245	15,557
Xc422	JAUBMC020000040.1	79.40	199	31,909	32,099
Xc422	JAUBMC020000040.1	74.41	801	8,392	9,178
Xc43	JAUBNN020000033.1	98.32	119	37,225	37,343
Xc43	JAUBNN020000033.1	97.48	119	2,955	3,073
Xc43	JAUBNN020000033.1	95.88	97	37,230	37,325
Xc43	JAUBNN020000033.1	95.35	129	2,314	2,442
Xc43	JAUBNN020000033.1	95.28	127	2,947	3,073
Xc43	JAUBNN020000033.1	94.85	97	2,319	2,414
Xc43	JAUBNN020000033.1	94.12	1,122	37,225	38,346

Xc43	JAUBNN020000033.1	91.51	212	3	208
Xc43	JAUBNN020000033.1	90.81	979	21,826	22,802
Xc43	JAUBNN020000033.1	90.70	4,044	22,875	26,916
Xc43	JAUBNN020000033.1	90.52	232	3,099	3,330
Xc43	JAUBNN020000033.1	88.83	1,137	4,976	6,107
Xc43	JAUBNN020000033.1	88.05	7,641	10,809	18,418
Xc43	JAUBNN020000033.1	86.02	7,826	27,469	35,246
Xc43	JAUBNN020000033.1	85.41	329	39,574	39,901
Xc43	JAUBNN020000033.1	85.33	593	6,226	6,802
Xc43	JAUBNN020000033.1	85.30	1,299	1,776	3,056
Xc43	JAUBNN020000033.1	84.09	886	9,412	10,289
Xc43	JAUBNN020000033.1	84.05	1,329	19,792	21,104
Xc43	JAUBNN020000033.1	83.85	1,932	7,203	9,118
Xc43	JAUBNN020000033.1	83.03	1,367	3,643	4,978
Xc43	JAUBNN020000033.1	78.89	199	37,464	37,654
Xc43	JAUBNN020000033.1	74.78	801	13,939	14,725
Xc433	JAUBMB020000033.1	98.32	119	31,329	31,447
Xc433	JAUBMB020000050.1	98.28	232	2,672	2,903
Xc433	JAUBMB020000050.1	96.70	91	1,401	1,490
Xc433	JAUBMB020000050.1	96.12	129	1,395	1,523
Xc433	JAUBMB020000033.1	95.88	97	31,334	31,429
Xc433	JAUBMB020000050.1	95.00	120	2,026	2,145
Xc433	JAUBMB020000048.1	94.62	130	3,999	4,128
Xc433	JAUBMB020000033.1	94.15	1,025	31,329	32,353
Xc433	JAUBMB020000050.1	92.65	136	2,018	2,153
Xc433	JAUBMB020000033.1	90.93	4,045	16,987	21,029
Xc433	JAUBMB020000033.1	90.81	979	15,938	16,914
Xc433	JAUBMB020000062.1	90.18	275	2,091	1,824
Xc433	JAUBMB020000033.1	87.77	7,634	4,928	12,530
Xc433	JAUBMB020000033.1	87.62	218	2	213
Xc433	JAUBMB020000033.1	86.20	1,768	341	2,092
Xc433	JAUBMB020000033.1	86.16	7,824	21,582	29,359
Xc433	JAUBMB020000050.1	85.28	2,343	3,222	5,530
Xc433	JAUBMB020000033.1	84.80	1,474	2,876	4,344
Xc433	JAUBMB020000050.1	84.40	1,718	857	2,529
Xc433	JAUBMB020000033.1	84.12	1,329	13,904	15,216
Xc433	JAUBMB020000033.1	79.40	199	31,568	31,758
Xc433	JAUBMB020000033.1	74.66	801	8,051	8,837
Xc442	JAUBMA020000035.1	94.15	188	41,250	41,063
Xc442	JAUBMA020000035.1	88.85	574	43,300	42,728
Xc442	JAUBMA020000035.1	84.52	1,098	35,922	37,009

Xc442	JAUBMA020000035.1	82.67	531	37,645	38,169
Xc442	JAUBMA020000035.1	82.53	372	3,069	3,431
Xc442	JAUBMA020000035.1	76.10	1,042	1,944	2,944
Xc452	JAUBLZ020000065.1	95.53	179	385	563
Xc452	JAUBLZ020000072.1	89.00	582	421	1,001
Xc452	JAUBLZ020000069.1	88.85	574	652	1,224
Xc452	JAUBLZ020000046.1	84.52	1,098	3,359	2,272
Xc452	JAUBLZ020000052.1	82.92	363	11,898	11,545
Xc452	JAUBLZ020000046.1	82.58	534	1,636	1,109
Xc452	JAUBLZ020000052.1	76.02	1,055	13,032	12,020
Xc58	JAUBNJ020000033.1	100.00	28,477	28,477	1
Xc58	JAUBNJ020000033.1	100.00	120	5,324	5,205
Xc58	JAUBNJ020000033.1	100.00	120	37,737	37,618
Xc58	JAUBNJ020000033.1	99.99	10,286	38,762	28,478
Xc58	JAUBNJ020000033.1	96.74	92	5,319	5,229
Xc58	JAUBNJ020000033.1	96.74	92	37,087	36,996
Xc58	JAUBNJ020000033.1	96.74	92	37,087	36,996
Xc58	JAUBNJ020000033.1	96.74	92	37,732	37,642
Xc7	JAUBMR020000038.1	100.00	40	5,066	5,027
Xc7	JAUBMR020000038.1	99.16	119	6,729	6,611
Xc7	JAUBMR020000038.1	98.39	62	5,066	5,005
Xc7	JAUBMR020000029.1	98.32	119	4,526	4,408
Xc7	JAUBMR020000038.1	97.10	69	5,066	4,998
Xc7	JAUBMR020000038.1	96.74	92	6,724	6,634
Xc7	JAUBMR020000038.1	96.43	644	5,026	4,383
Xc7	JAUBMR020000029.1	95.88	97	4,521	4,426
Xc7	JAUBMR020000038.1	94.62	130	13,086	12,957
Xc7	JAUBMR020000029.1	94.15	1,025	4,526	3,502
Xc7	JAUBMR020000029.1	91.16	3,926	19,084	15,161
Xc7	JAUBMR020000029.1	90.07	987	20,142	19,156
Xc7	JAUBMR020000038.1	88.08	1,032	7,047	6,025
Xc7	JAUBMR020000038.1	87.97	582	9,098	8,519
Xc7	JAUBMR020000029.1	87.84	7,630	31,995	24,394
Xc7	JAUBMR020000029.1	87.70	374	37,076	36,709
Xc7	JAUBMR020000029.1	86.04	1,784	36,581	34,815
Xc7	JAUBMR020000038.1	85.54	2,352	4,070	1,753
Xc7	JAUBMR020000029.1	84.80	1,474	34,047	32,579
Xc7	JAUBMR020000029.1	84.75	7,883	14,322	6,496
Xc7	JAUBMR020000029.1	84.01	1,332	23,012	21,697
Xc7	JAUBMR020000029.1	79.40	199	4,287	4,097
Xc7	JAUBMR020000029.1	74.75	804	28,858	28,069

XRE1	JAUBLI020000046.1	94.62	130	4,931	4,802
XRE1	JAUBLI020000057.1	89.08	229	23	251
XRE1	JAUBLI020000046.1	87.33	521	4,618	4,099
XRE1	JAUBLI020000039.1	86.95	590	2,102	1,532
XRE1	JAUBLI020000039.1	83.25	197	4,084	3,888
XRE5	JAUBLH020000051.1	94.62	130	4,931	4,802
XRE5	JAUBLH020000062.1	89.08	229	23	251
XRE5	JAUBLH020000051.1	87.33	521	4,618	4,099
XRE5	JAUBLH020000043.1	86.95	590	2,102	1,532
XRE5	JAUBLH020000043.1	83.25	197	4,084	3,888
